# To Test the Constitutionality of the Texas Medical Practice Law

**Published:** 1904-11

**Authors:** 


### THE
### TEXAS MEDICAL JOURNAL.

AUSTIN, TEXAS.

A MONTHLY JOURNAL OF MEDICINE AND SURGERY.

EDITED AND PUBLISHED BY
F. E. DANIEL, M. D.
Mrs. F. E. DANIEL, Managing Editor.

Published Monthly at Austin, Texas. Subscription price $1.00 a year in advance.

Eastern Representative: John Guy Monihan, St. Paul Building, 220 Broadway New York City.

Official organ of the West Texas Medical Association, the Houston District Medical Association, the Austin District Medical Society, the Brazos Valley Medical Association, the Galveston County Medical Society, and several others.

To Test the Constitutionality of the Texas Medical Practice Law.—A case from Jackson county, Texas, is on appeal before the Court of Civil Appeals at Austin to test the constitutionality of our medical practice law. A decision is awaited with great interest by the medical profession, and especially by the State Association's Committee on Public Policy and Legislation. That committee and representatives from many county medical societies will meet in Austin November 14th, and, before this is in type, they will have been in session. One object of the meeting is to discuss amendments to the medical practice act, and amendments will be asked of the approaching Legislature to remedy the defects, which it is claimed render the law unconstitutional. The Constitution says that "the State shall have the power to regulate the practice of medicine, but no preference shall be shown any school of medicine." The Texas law "shows preference" to all who "do not give drugs or medicines," by exempting them from the requirements imposed upon all other practitioners before permitting them to practice; and hence the osteos, the magnetic healers, the vito-paths, the Christian Scientists *et id. om. gen.* are allowed to "practice medicine" without examination or license. The courts in several States have decided that the "drugless terrors," as I call them, are *practicing medicine* in the meaning of the law.

One Stone in Jackson county was indicted for practicing medicine in violation of the law, and was convicted. His lawyer took an appeal, alleging that the law is unconstitutional, as it clearly is, in the opinion of yours truly.

